# Serum hyaluronate as a non-invasive marker of hepatic fibrosis and inflammation in HBeAg-negative chronic hepatitis B

**DOI:** 10.1186/1471-230X-5-32

**Published:** 2005-10-12

**Authors:** Ghodrat Montazeri, Arezoo Estakhri, Mehdi Mohamadnejad, Negin Nouri, Farhad Montazeri, Ashraf Mohammadkani, Mohammad Hossain Derakhshan, Farhad Zamani, Shahram Samiee, Reza Malekzadeh

**Affiliations:** 1 Digestive Disease Research Centre, Tehran University of Medical Science, Tehran, Iran; 2 Gastrointestinal and Liver Disease Research Center, Iran University of Medical Sciences, Tehran, Iran

## Abstract

**Background:**

HBV infection is a serious global heath problem. It is crucial to monitor this disease more closely with a non-invasive marker in clinical trials. We aimed to evaluate the predictive value of serum hyaluronate for the presence of extensive liver fibrosis and inflammation.

**Methods:**

28 healthy volunteers and 65 patients with HBeAg negative chronic hepatitis B were enrolled. Liver biopsies scored according to Ishak system. Association of serum hyaloronate with liver fibrosis and inflammation were assessed, and cut off points for serum hyaluronate levels were identified by receiver operating characteristics (ROC) curves and their values for prediction of fibrosis and inflammation were assessed.

**Results:**

In patients with CHB serum hyaluronate had the most significant correlation and predictive values for the liver fibrosis and inflammation comparing to the other variables. At the cut off point of 126.4 ngm/ml it could discriminate extensive fibrosis from milder ones with sensitivity of 90.9% and specificity of 98.1%. With the same value it could discriminate extensive inflammation from their milder counterparts with sensitivity of 63.6% and specificity of 92.6%.

**Conclusion:**

Serum hyaluronate was the best predictor of extensive liver fibrosis and inflammation and it could discriminate subgroups of patients with chronic hepatitis B. It could be used as a non-invasive test to monitor these patients more closely with developing anti viral agents in clinical trials.

## Background

HBV infection is a serious global health problem. Of the 2 billions people who have been infected with HBV, more than 350 millions are chronically infected world wide [1,2]. Chronic HBV infection is the major cause of end stage liver disease, 25% of them will die prematurely of liver cirrhosis or hepatocellular carcinoma [3]. It is crucial to monitor the course of this disease more closely, especially with developing antiviral agents in clinical trials [4]. Liver biopsy is currently considered the gold standard for evaluation of liver fibrosis [5]. However it has several limitations like sampling error, post biopsy pain and death [6-9]. It is expensive procedure and could be exceedingly dangerous in cases of advanced liver disease with prolonged prothrombin time and low platelet count [10]. Liver function tests are essential parts of assessing liver damage, but have poor correlation with histology [11-13]. Because of limitations in conventional approaches, several non invasive tests have been developed for this purpose. Among them, serum hyaluronate as a direct marker of liver fibrosis appears to be the most promising one [14-17]. The studies have shown that hyaluronic acid increase in acute liver failure [18], primary billiary cirrhosis [19], alcoholic liver disease [20] and chronic hepatitis C [15].

Most of those studies have been performed in patients with chronic hepatitis C. The value of such markers in patients with in chronic hepatitis B remained unclear. We aimed to find out the utility of serum hyaluronate to evaluate the presence of extensive liver fibrosis and inflammation in patients with chronic hepatitis B.

## Methods

### Study population

Population groups under study consist of 28 normal volunteers, 65 patients with HBeAg negative chronic hepatitis B. We defined chronic hepatitis B as subjects who met at least two of three following criteria: 1) serum level of aminotransferases above 1.5–10 times of upper limit of normal. 2) total score of 4 or more according to Ishak (modified HAI) scoring system, and 3) viral DNA load of more than 1.77 pg/ml (equivalent to 500,000 copies/ml) using a signal amplification technique (Naxcor assay). Patients of chronic hepatitis B were selected from an open labelled Lamivudine trial from January 2001 to January 2004. All patients with chronic hepatitis B were negative for HBe antigen. They were all negative for HDV Ab, HCV Ab and HIV Ab. None of them had any evidence for decompensated cirrhosis or any other cause for chronic liver disease. Control group were selected from healthy volunteers. Five ml of peripheral of blood was taken from each patient in fasting state, one hour before performing liver biopsy and in complete rest. serum was isolated and kept at -70 degrees centigrade for measurement of serum hyaluronate. The same procedure was applied for normal volunteers.

### Assessment of liver histology

Liver biopsy was performed by automatic true cut biopsy needle. Length and width of each sample were at least 10 mm and 1.4 mm respectively and contained 4 or more portal spaces. The liver histology was scored according to Ishak (modified HAI) scoring system [21]. Maximum grade (inflammation) was scored 18, and maximum stage (fibrosis) was scored 6. Stage 3 or more was considered as significant fibrosis, and stage 5 or 6 was considered as cirrhosis. All biopsies were reviewed by a single pathologist who was unaware of patients' clinical records.

### Measurement of hyaluronic acid

Hyaluronic acid test kit was provided by Corgenix Inc. (Colorado, USA, under licence of Chugai diagnostic science Co.). Serum hyaluronate was measured by ELISA according to instruction manual of manufacturer. All samples used in duplicate. Briefly 100 μl of serum or reference solution (diluted 1:10) was added to each HABP (hyaluronic acid binding protein) coated micro well, incubated for 60 minutes, after washing 100 μl HRP-conjugated-HABP solution was added and incubated for 30 minutes. Eventually 100 μl of stopping solution (0.36 N sulphuric acid) was added. Optical density (OD) was read at 450 nm. Water was used as zero blank. Standard curve was produced by using ODs of 50,100, 200, 500, and 800 ng/ml of reference solution. Linear regression equation of standard solutions was produced. Values of all samples were computed on the basis of regression equation of standard solutions.

### Statistical considerations

Statistical analyses were performed by using the SPSS, version 10.1, software package (SPSS, Inc., Chicago, IL). Data are reported as means ± standard deviation (SD). Patients with CHB were arbitrary divided into subgroups according to the degrees of fibrosis (stage 2 or less vs stage 3 or more) and inflammation (grade 0–8 vs grade 9 or more). The associations between factors (age, AST, ALT, alkaline phosphatase, platelet count, albumin, total bilirubin, prothrombin time (PT), and serum hyaluronate) with liver fibrosis or inflammation were assessed by Spearman correlation test. Then, independent factors associated with extensive fibrosis or inflammation were assessed by the regression analysis. Subsequently, the sensitivity, specificity, positive predictive value (PPV) and negative predictive value (NPV) of hyaluronate to differentiate between subgroups of patients with chronic hepatitis was calculated using receiver operating characteristics (ROC) curve with different cut-off points. P values less than 0.05 were considered statistically significant.

## Results

Twenty eight normal volunteers consist of 17 male and 11 female with the mean age of 32.0 ± 5.2 (range: 20–44) years old. Sixty five patients with chronic hepatitis B, consist of 54 male and 11 female with the mean age of 39.8 ± 12.3 (range: 18 – 76) years old. All of them had HBeAg negative CHB. Fifty four out of 65 patients with chronic hepatitis B had mild fibrosis (stage 0–2) and 11 (17%) had extensive fibrosis (stage 3–5).

Serum hyaluronate levels of the populations under study were shown in table 1. Normal volunteers had mean ± SD of serum hyaluronate level of 20.4 ± 15.4 ng/ml. The upper limit of normal (as defined by the 95^th ^percentile of the variable) in the normal volunteers was 58.6 ng/ml. Patients with chronic hepatitis B as a single group had higher serum hyaluronate level than normal volunteers (73.4 ± 124.8 vs 20.4 ± 15.5 ng/ml respectively; P = 0.002). Serum hyaluronate level of cases with extensive fibrosis was significantly higher than those with mild fibrosis (309.7 ± 143.5 vs 24.7 ± 31.9 respectively; p < 0.001).

Fifty four out of 65 of CHB patients with mild inflammation (grade 0–8) had hyaluronate level of 39.6 ± 74.2 ngm/ml, while eleven of them with severe inflammation (grade = 9) had serum hyaluronate level of 236.5 ± 188.7 ngm/ml (p = 0.006) (Table 1).

**Figure 1:**
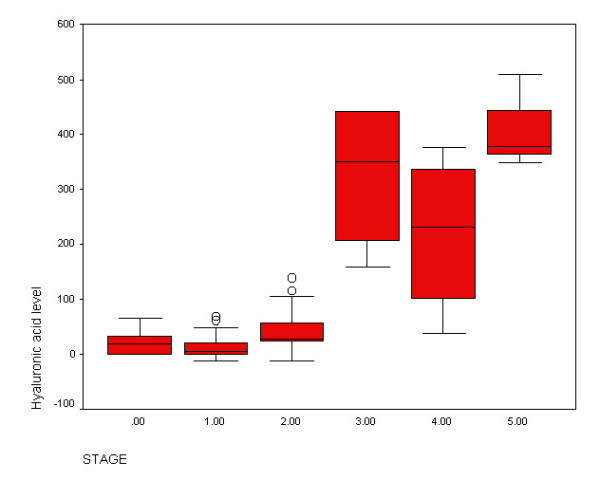
Box plot of serum hyaluronate level in relation to the Ishak fibrosis score. The box represents the interquartile range, the whiskers indicate the highest and lowest values, and the circles represent outliers. The line across the box indicates the median value.

Correlation of the variables with the level of fibrosis, and inflammation were assessed by Spearman correlation test. Serum hyaluronate had the most significant correlation with the level of fibrosis comparing to the other variables (Correlation coefficient = 0.58, p < 0.001) (Figure 1).

Next to that was correlation of serum hyaluronate to the level of inflammation which was highly significant comparing to the remaining variables (correlation coefficient = 0.42, p < 0.001). (Table 2).

Logistic regression analysis was carried out to find the independent variables associated with extensive liver fibrosis and inflammation in two separate analyses. Stage and grade were selected as dependent factor and other variables (age, hyaluronic acid, AST, ALT, alkaline phosphatase, platelet count, albumin, total billirubin and PT) as independent factors. Serum hyaluronate was the only independent factor associated with extensive liver fibrosis (P = 0.005), and inflammation (P < 0.001) in patients with chronic hepatitis B.

**Figure 2:**
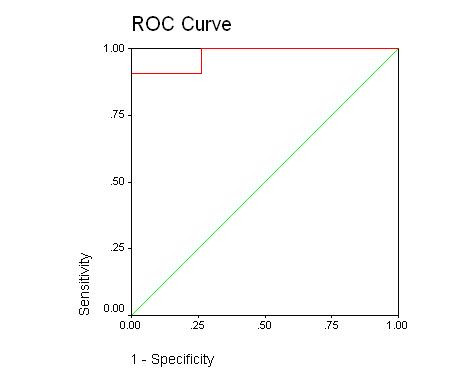
Receiver operating characteristics curve (ROC) of serum hyaluronate for discrimination of mild (stage 0–2) from significant (stage 3 or more) of fibrosis. Hyaluronate at cut off point of 126.43 ngm/ml could differentiate mild from severe fibrosis in patients with chronic hepatitis B.

**Figure 3:**
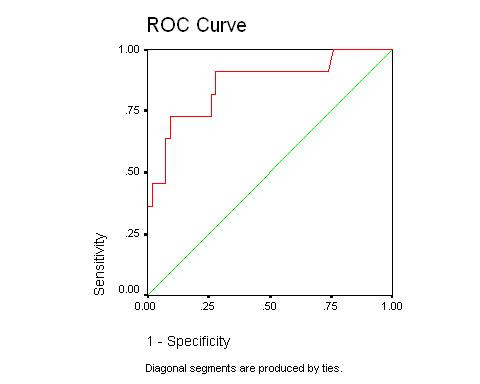
Receiver operating characteristics curve (ROC) of hyaluronate for discrimination of mild (grade 0–8) from significant (grade ≥ 9) inflammation. Hyaluronate at cut off point of 126.43 ngm/ml could differentiate mild from severe inflammation in patients with chronic hepatitis B.

The ability of serum hyaluronate as a non-invasive test to discriminate levels of fibrosis and inflammation was assessed by ROC curve. Within the subgroups of chronic hepatitis B, serum hyaluronate at the cut-off point of 126.4 ngm/ml could discriminate mild (stage 0–2) from extensive fibrosis (stage 3–5) with an area under ROC curve (AUC) of 0.98, sensitivity of 90.9%, specificity of 98.1%, PPV of 90.9% and NPV of 98.1% (Figure 2). On the same basis serum hyaluronate could differentiate mild (grade 0–8) from extensive inflammation (grade ≥ 9) with an AUC of 0.86, the sensitivity of 63.6%, specificity of 92.6%, PPV of 63.6% and NPV of 92.6% (Figure 3).

## Discussion

In this study we have shown that::1) Sub groups of patients with HBeAg negative chronic hepatitis B who had extensive fibrosis and inflammation had also high serum hyaluronate level. The differences were statistically significant comparing to normal values and to the patients with milder liver involvement. 2) Serum hyaluronate had more significant correlation with severity of liver fibrosis and inflammation amongst the other variables. 3) In the regression analysis serum hyaluronate had best predictive value for liver fibrosis and inflammation in patients with chronic hepatitis B. 4) At cut-off point of 126.4 ngm/ml it could differentiates extensive fibrosis from milder ones with sensitivity of 90.9% and specificity of 98.1%. At the same cut-off point, it could differentiate extensive inflammation from milder ones with sensitivity of 63.6% and specificity of 92.6%.

Liver biopsy is the method of choice in evaluating fibrosis and inflammation in patients with parenchymal liver disease [5,17], but it has several limitations which include false negative result of 24% especially in macro nodular cirrhosis [6,7], post biopsy pain and discomfort [9], high cost [10], and death rate of 0.015% [8]. In the past decade, several investigators focused on developing non-invasive test for liver fibrosis [16,17,22]. None of them proved to be perfect. Guechot et al showed that hyaluronate had better correlation to the degrees of fibrosis than PIIIP in chronic liver disease [23]. In the study by Oberti et al, serum hyaluronate level was considered the most sensitive test for screening in viral hepatitis B, and C [14]. Imbert-Bismuth et al suggested that, by fibro test 50% of liver biopsies could be avoided in patient with chronic hepatitis C [24]. However fibro test was reanalysed in treated hepatitis C, but its complex equation will limit its usefulness in clinical practice [25]. Thus, due to expense and complexity of Fibrotest-Actitest, it could not be utilized on routine basis in developing countries. Myers et al used Fibrotest and Actitest to decriminate.mild from extensive fibrosis and inflammation in patients with chronic hepatitis B [26]. In Spite of the interesting results, it is difficult to compare it with our data. However, hyaluronic acid may have a lower sensitivity for minimal fibrosis (as in chronic hepatitis C) as well as an absence of independent assessment of both fibrosis and activity as given by FibroTest-Actitest. We were looking for a direct marker of fibrogenesis to be cheap, reproducible and simple. The price of hyaluronic acid test is about 10 US Dollars in Iran as well as in Europe. In this regard serum hyaluronate appears to be an appropriate choice.

Hyaluronate is a polysaccharide with molecular weight ranging from 4 × 10^3 ^to 8 × 10^6 ^Daltons. It forms constituent of extracellular matrix in all connective tissues [27-29]. It is mainly produced by mesenchymal cells and cleared by hepatic sinusoidal endothelial cells through a high affinity receptor (Kid = 6 × 10^-11 ^M) with a maximum capacity of 10^4 ^molecules/cells [30-33] It has short half life and increases by age [18].

Alcohol, viruses, auto immune diseases, and inborn errors of metabolism could increase production of hyaluronate by activating hepatic stellate cells and decrease clearance by hepatic sinusoidal capilarization [34]. Sinusoidal capilarization could be associated with shunting of blood which is an additional factor for increase of serum hyaluronate in this condition [35]. It was shown that serum hyaluronate increase in alcoholic liver disease [5,20,35], primary billiary cirrhosis [19] and in patients with hepatitis C [12,36]. In addition, it could be increased in rheumatoid disease due to overproduction by synovial cells [34,38]. It also increases in renal failure because of disturbed clearance of low molecular weight hyaluronate by the kidneys [39].

Aetiology of liver damage in our patients was only HBV infection. None of them had received any alcohol in their life time. None of them had any evidence for renal failure or any other disease which could explain their liver disease except HBV. Bloods were taken in complete physical rest and fasting state in order to rule out other interfering factors like eating and physical activity. Our normal level was 20.4 ± 15.5 ngm/ml which is in agreement with manufacturer levels (28.5 ± 24) and what we reported by the other investigators with the same age groups [15,16,20,40].

The proposed cut-off points in different studies were not the same. The cut-off point will depend on the aetiology of liver disease and on the level of sensitivity and specificity that an investigator is looking for [14,15,23]. Our study showed that the cut-off point of 126.4 ngm/ml could differential extensive fibrosis from milder ones with sensitivity of 90.9% and specificity of 98.1% and at the same level could differentiate extensive inflammation from milder ones with sensitivity of 63.6% and specificity of 92.6% in patients with chronic hepatitis B. Also, it should be noted that the cut-off value for a given variable depends upon the sample in which it has been identified. In order to be reproducible, a cut-off value should be obtained in a sample representative of the population with the disease.

Interestingly enough our results showed that, serum hyaluronate had best predictive value for the fibrosis and inflammation comparing to the other variables. This is in agreement with report of Ding H et al in which serum hyaluronate reflect both inflammation and fibrosis in HBV infection [41]. Currently it is believed that serum hyaluronate is a marker of liver fibrosis rather than inflammation. To what extent increased level of hyaluronate is due to inflammation alone without fibrosis, is difficult question to answer at the moment. It is because of complex interrelation of fibrogenesis and inflammatory process in vivo, which makes separation of pure fibrosis and inflammation impossible in a clinical setting. Elucidation of this complex issue requires further work. As stated above, regression analysis showed that only serum hyaluronate was associated with significant liver fibrosis. It should not be misinterpreted that other important factors (e.g. age, serum albumin, etc) are not associated with liver fibrosis in CHB. Since, the association of such a powerful factor (e.g. serum hyaluronate) was so close to the outcome (liver fibrosis) that the effects of other factors were excluded from the regression model.

Our study has some limitations. First and the most important limitation is that our study is considered a training study, and our data should be validated in another set of patients with chronic hepatitis B. Secondly, number of our patients with extensive fibrosis was relatively small. Further works are underway to validate this test, and also to find out other markers of liver fibrosis and inflammation in patients infected with hepatitis B virus.

## Conclusion

In conclusion our data indicated that serum hyaluronate had significant correlation and predictive value for the presence of significant liver fibrosis and inflammation comparing to the other variables. In addition it was able to discriminate extensive fibrosis and inflammation from their milder counterparts in patients with HBeAg negative chronic hepatitis B with good sensitivity and specificity. We think serum hyaluronate is a useful non-invasive test to monitor these patients more frequently in clinical trials.

## List of abbreviations

HBV: Hepatitis B virus

CHB: Chronic hepatitis B

ROC: Receiver Operating Characteristics

AUC: area under ROC curve

HBeAg: Hepatitis B e Antigen

ALT: Alanine aminotransferase

TGF-β: Transforming growth factor Beta

HDV: Hepatitis D Virus

HCV: Hepatitis C Virus

HABP: hyaluronic acid binding protein

OD: Optical Density

PPV: Positive Predictive Value

NPV: Negative Predictive Value

## Competing interests

This study was supported by local funds from digestive disease research center, Tehran University of medical sciences and has had no external financial support

## Authors' contributions

GM has been involved in the design and conception of the study, supervision of the work, and writing the manuscript. AE, NN, FM, SS contributed in the design of the study, and acquisition and analysis of data. MM carried out liver biopsies, and contributed in analysis and interpretation of data, and drafting the manuscript. AM carried out all laboratory assays. MHD contributed in analysis, and interpretation of data, and writing the manuscript. FM carried out liver biopsies and contributed in acquisition of data. RM contributed in the design of the study, supervised the study, and contributed in drafting of the manuscript. All authors read and approved the final manuscript.

## Pre-publication history

The pre-publication history for this paper can be accessed here:



## Figures and Tables

**Table 1:** Level of Hyaluronate in subgroup of chronic hepatitis B comparing to normal control

**Patient Groups**	**Subgroups**	**n**	**Mean ± SD**	P value*
Normal control		28	20.4 ± 15.4	
Chronic hepatitis B subgroups (degree of fibrosis)	Stage 0–2	54	24.7 ± 31.9	0.69
	Stage 3–5	11	309.7 ± 143.5	<0.001
Chronic hepatitis B subgroups (degree of inflammation)	Grade 0–8	54	39.6 ± 74.2	0.10
	Grade ≥ 9	11	236.5 ± 188.7	0.003

*Statistical difference between serum hyaluronate of different groups compared with normal controls.

**Table 2:** Correlation between factors and stages of liver fibrosis on Spearman correlation test

**Factor**	**Correlation coefficient**	**P value**
Age	0.27	0.03
PT	0.30	0.02
Platelet counts	- 0.24	0.05
AlkP	0.44	<0.001
Serum Hyaluronate	0.58	<0.001
Serum Albumin	- 0.44	<0.001
Total Bilirubin	0.17	0.19
AST	- 0.04	0.73
ALT	- 0.12	0.36

**Abbreviations: **PT: Prothrombin time; AST: Serum aspartate aminotransferase; ALT: Serum alanine aminotransferase; AlkP: Serum alkaline phosphatase.

## References

[B1] Lee WM (1997). Hepatitis B virus infection. N Engl J Med.

[B2] Kane M (1995). Global programme for control of hepatitis B infection. Vaccine.

[B3] Mast EE, Alter MJ (1993). Epidemiology of viral hepatitis: an overview. Sem Virol.

[B4] Dienstag JL, Goldin RD, Heathcote EJ, Hann HW, Woessner M, Stephenson SL, Gardner S, Gray DF, Schiff ER (2003). Histological outcome during long-term lamivudine therapy. Gastroenterology.

[B5] Phillips MG, Preedy VR, Hughes RD (2003). Assessment of prognosis in alcoholic liver disease: can serum hyaluronate replace liver biopsy?. Eur J Gastroenterol Hepatol.

[B6] Nord HJ (1982). Biopsy diagnosis of cirrhosis: blind percutaneous versus guided direct vision techniques–a review. Gastrointest Endosc.

[B7] Maharaj B, Maharaj RJ, Leary WP, Cooppan RM, Naran AD, Pirie D, Pudifin DJ (1986). Sampling variability and its influence on the diagnostic yield of percutaneous needle biopsy of the liver. Lancet.

[B8] Piccinino F, Sagnelli E, Pasquale G, Giusti G (1986). Complications following percutaneous liver biopsy. A multicentre retrospective study on 68,276 biopsies. J Hepatol.

[B9] Cadranel JF, Rufat P, Degos F (2000). Practices of liver biopsy in France: results of a prospective nationwide survey. For the Group of Epidemiology of the French Association for the Study of the Liver (AFEF). Hepatology.

[B10] Quinn PG, Johnston DE (1997). Detection of chronic liver disease: costs and benefits. Gastroenterologist.

[B11] Hayes PC, Bouchier IA (1989). Liver function test in clinical practice: Their uses and limitations. Clin Chem Enzym Commun.

[B12] Chopra S, Griffin PH (1985). Laboratory tests and diagnostic procedures in evaluation of liver disease. Am J Med.

[B13] Hayes PC, Thomas P, Bouchier IA, Piris J (1990). Biochemistry and histology in chronic liver disease. Clin Chem Enzym Commun.

[B14] Oberti F, Valsesia E, Pilette C, Rousselet MC, Bedossa P, Aube C, Gallois Y, Rifflet H, Maiga MY, Penneau-Fontbonne D, Cales P (1997). Non-invasive diagnosis of hepatic fibrosis and cirrhosis. Gastroenterology.

[B15] Plevris JN, Haydon GH, Simpson KJ, Dawkes R, Ludlum CA, Harrison DJ, Hayes PC (2000). Serum hyaluronate- a non-invasive test for diagnosis liver cirrhosis. Eur J Gastroenterol Hepatol.

[B16] Croquet V, Vuillemin E, Ternisien C, Pilette C, Oberti F, Gallois Y, Trossaert M, Rousselet MC, Chappard D, Cales P (2002). Prothrombin index is an indirect marker of severe liver fibrosis. Eur J Gastroenterol Hepatol.

[B17] Cadranel JF, Mathurin P (2002). Prothrombin index decrease: a useful and reliable marker of extensive fibrosis?. Eur J Gastroenterol Hepatol.

[B18] Bramley PN, Rathbone BJ, Forbes MA, Cooper EH, Losowsky MS (1991). Serum hyaluronate as a marker of hepatic derangement in acute liver damage. J Hepatol.

[B19] Nyberg A, Engstrom-Laurent A, Loof L (1988). Serum hyaluronate in primary billiary cirrhosis- a biochemical marker for progressive liver disease. Hepatology.

[B20] Stickel F, Poseschl G, Schuppan D, Conradt C, Srrenge-Hesse A, Fuchs FS, Hofmann WJ, Seitz HK (2003). Serum hyaluronate correlates with histological progression in alcoholic liver disease. Eur J Gatroenterol Hepatol.

[B21] Ishak K, Baptista A, Bianchi L, Callea F, Groote JD, Guadat F, Denk H, Desmet V, Korb G, MacSween RN, Phillips MJ, Portmann BG, Poulsen H, Scheuer PJ, Schmid M, Thaler H (1995). Histological grading and staging of chronic hepatitis. J Hepatol.

[B22] Wai CT, Greenson JK, Fonata RJ, Kalbflisch JD, Marrero JA, Conjeevaram HS, Lok AS (2003). A simple non-invasive index can predict both significant fibrosis and cirrhosis in patients with chronic hepatitis B. Hepatology.

[B23] Guechot J, Poupon RE, Giral P, Balkau B, Giboudeau J, Poupon R (1994). Relationship between procollagen III aminoterminal propeptide and hyaluronan serum levels and histological fibrosis in primary biliary cirrhosis and chronic viral hepatitis C. J Hepatol.

[B24] Imbert-Bismuth F, Ratziu V, Pieroni L, Charlotte F, Benhamou Y, Poynard T (2001). Biochemical markers of liver fibrosis in patients with hepatitis C virus infection: a prospective study. Lancet.

[B25] Poynard T, Imbert-Bismut F, Ratizu V, Chevret S, Jardel J, Mossalli J (2002). Biochemical markers of liver fibrosis in patients infected by hepatitis C virus: longitudinal validation in a randomized trial. J Viral Hepat.

[B26] Myers RP, Tainturier MH, Ratziu V, Piton A, Thibault V, Imbert-Bismut F, Messous D, Charlotte F, Di Martino V, Benhamou Y, Poynard T (2003). Prediction of liver histological lesions with biochemical markers in patients with chronic hepatitis B. J Hepatol.

[B27] Meyer K, Plmer JW (1934). The polysaccharide of the vitrous humor. J Biol Chem.

[B28] Laurent TC, Fraser JRE (1986). The properties and turnover of hyaluronate. Ciba Foundation Symposium 124, ed Functions of proteoglycans.

[B29] Tamaki S, Ueno T, Torimura T, Sata M, Tanikawa K (1996). Evaluation of hyaluronic acid binding ability of hepatic sinusoidal cells in rat liver with liver cirrhosis. Gastroenterology.

[B30] Smedsrod B, Pertoft H, Erikson S, Fraser RF, Laurent TC (1984). Studies in vitro on the uptake and degradation of sodium hyaluronate in rat liver endothelial cells. Biochem J.

[B31] Truppe W, Basner R, Von Figura K, Kresse H (1977). Uptake of hyaluronate by cultured cells. Biochem Biophys Res Commun.

[B32] Fraser JR, Laurent TC, Engstrom-Laurent A, Laurent UB (1984). Elimination of hyaluronic acid from the blood stream in the human. Clin Exp Phrmacol Pyhsiol.

[B33] Tamaki S, Ueno T, Torimura T, Sata M, Tanikawa K (1996). Evaluation of hyaluronic acid binding ability of hepatic sinusoidal cells in rat liver with liver cirrhosis. Gastroenterology.

[B34] Ueno T, Inuzuka S, Torimura T, Tamaki S, Kou H, Kin M, Minetoma T, Kimura Y, Ohira H, Sata M, Yoshida H, Tanikawa K (1993). Serum hyaluronate reflects hepatic sinusoidal capilarization. Gastroenterology.

[B35] Gibson PR, Fraser JR, Brown JT, Finch CF, Jones PA, Colman JC, Dudley FJ (1992). Hemodynamic and liver function predictors of serum hyaluronate in alcoholic liver disease. Hepatology.

[B36] Kojima H, Hongo Y, Harada H, Inouce T, Miyaji K, Kashiwagi M, Momose T, Arisaka Y, Fukui H, Murai S, Tokita H, Kamitsukasa H, Yagura M, Katsu K (2001). Long-term histological prognosis and serum fibrosis marker in chronic hepatitis C patients treated with interferon. J Gastroenterol Hepatol.

[B37] Gressner AM, Schafer S (1989). Comparison of sulphated glycosaminoglycan and hyaluronate synthesis and secretion in cultured hepatocyte, fat storing cells and kupffer cells. J Clin Chem Clin Biochem.

[B38] Engstrom-Laurent A, Feltelius N, Hallgren R, Wasteson A (1985). Raised serum hyaluronate levels in scleroderma: an effect of growth factor induced activation of connective tissue cells. Ann Rheum Dis.

[B39] Hallengen R, Engstrom-Laurent A, Nisbeth U (1982). Circulating hyaluronate potential marker of altered metabolism of the connective tissue in uremia. Nephron.

[B40] Laurent UB, Laurent TC (1981). On the origin of serum hyaluronate in blood. Biochem Int.

[B41] Ding H, Chen Y, Feng X, Liu D, Wu A, Zhang L (2001). Correlation between liver fibrosis stage and serum liver fibrosis markers in patients with chronic hepatitis B. Zhonghua Gan Zang Bing Za Zhi.

